# Ophthalmological impairment in patients with congenital cytomegalovirus infection

**DOI:** 10.3389/fped.2023.1251893

**Published:** 2023-11-17

**Authors:** Serena Salomè, Nicola Ciampa, Mariapaola Giordano, Raffaele Raimondi, Eleonora Capone, Claudia Grieco, Clara Coppola, Letizia Capasso, Francesco Raimondi

**Affiliations:** ^1^ Department of Translational Medical Sciences—Division of Neonatology, University “Federico II”, Naples, Italy; ^2^ Department of Neurosciences, Reproductive and Odontostomatologic Sciences—Unit of Ophthalmology, University “Federico II”, Naples, Italy

**Keywords:** congenital CMV, cytomegalovirus, ocular outcome, chorioretinitis, ophthalmological impairment

## Abstract

**Background:**

Congenital cytomegalovirus (cCMV) infection is a frequent cause of neurosensory impairment. Ocular abnormalities and visual impairment have been reported in a high percentage of symptomatic infants, whereas they are considered uncommon in asymptomatic ones. The paucity of data has made difficult to reach clear recommendations on the ophthalmological follow-up that should be provided.

**Methods:**

250 patients with cCMV infection (123 symptomatic) were enrolled and underwent a series of age-appropriate ophthalmologic, audiologic, and neurodevelopmental examinations from 2002 to 2022.

**Results:**

Funduscopic abnormalities were identified at onset in 16/123 (13%) symptomatic infants and in none of the asymptomatic ones (*p* < 0.001). Chorioretinitis lesions were the most common findings (10/16 cases), while the others showed retinal scars. Lesions were bilateral in 4 patients. No later onset retinal lesions were detected, nor in symptomatic or in asymptomatic children. Five of the 16 (31.5%) symptomatic and none of the asymptomatic subjects showed visual impairment al the last evaluation (*p* < 0.001). All patients with unfavorable outcome had also neurological impairment. Among symptomatic patients, ocular lesions were associated with central nervous system (CNS) pathological findings in prenatal ultrasonography (*p* 0.05) and with clinical signs of CNS involvement at birth (*p* 0.046). No correlation was found with the type of maternal infection and pathological neuroimaging.

**Conclusions:**

Chorioretinal lesions are a fairly common finding at birth in neonates with symptomatic cCMV, often associated with long term visual impairment. Asymptomatic infants do not show ophthalmological abnormalities in the short or long term. This information is relevant both to parental counseling and to cost-effective patient management.

## 1. Introduction

Cytomegalovirus (CMV) is the most common cause of congenital viral infection (1). It affects approximately 0.64% of all live births although this varies considerably among different populations (2) and in Italy is 0.47% (0.22%–1.0%) (3). Approximately 10% of congenitally infected newborns present one or more clinically observable abnormalities at birth, which include microcephaly, jaundice, hepatosplenomegaly, petechiae, hearing loss, and chorioretinitis (4). Large follow-up studies have mainly focused on hearing and neurodevelopmental outcomes, while long-term ocular outcomes are not as well studied or as well known yet. A variety of clinical ophthalmological signs are associated with congenital CMV, such as chorioretinitis, optic atrophy, macular scars, strabismus, and cortical visual impairment (4–7). Ocular abnormalities and visual impairment have been reported in a large percentage (5%–30% of infants with CMV disease) of symptomatic infants with congenital CMV (cCMV) infection, whereas they are considered uncommon in otherwise asymptomatic infants [up to 1% in Coats K et al, (5)] (7, 8) or absent (9). Unlike SNHL, visual defects are not typically progressive or delayed in onset.

Visual impairment is described in about 20% of children with symptomatic cCMV. The most common sequelae of ophthalmological involvement at birth were chorioretinal scars (up to 49%), strabismus (from 19%–29% (5, 9) to 5.5 (8), cortical visual impairment, nystagmus, and optic nerve atrophy (5, 8, 9). However, cCMV can also present with an atypical retinal finding mimicking the retinopathy of prematurity, as recently reported in a term-born infant congenitally infected by CMV (10).

The paucity of data has made difficult to reach clear recommendations on the ophthalmological follow-up that should be provided to infants with cCMV infection (4) and if it should be different for symptomatic and asymptomatic children. The need of clear guidelines for ophthalmic surveillance was reported also by Karamchandani U et al. (11). The aim of this study was to evaluate ophthalmological findings and visual function in the neonatal period and during a long-term follow-up in infants with cCMV infection. The ocular involvement in relation to type of maternal infection was also explored.

## 2. Material and methods

### 2.1. Study population

This is an observational study conducted at the Perinatal Infection Unit of the University Federico II of Naples, a tertiary care hospital with a dedicated multidisciplinary team. All infants with cCMV infection referred to the Unit between January 2002 and December 2022 were enrolled. They were identified because of suspected/confirmed maternal CMV infection during pregnancy or because of the presence of symptoms consistent with cCMV infection at birth.

Congenital infection was defined as viral DNA detection in urine by polymerase chain reaction assay within the first 3 weeks of life (4). At enrollment time a blood sample was also collected to determine viremia. Viral DNA detection (both on urine and blood) was performed using an automated QIAsimphony SP platform performing all steps of the purification procedure and preparation for CMV DNA amplification, in combination with the Artus CMV QS-RGQ Kit on Rotor-Gene® Q system, accordingly to the manufacturer’s instructions.

Maternal CMV infections were categorized by analyzing maternal and newborn hospital records. In order to classify maternal CMV infection, we used the accepted criteria recently reviewed by Maltezou P-Georgia et al, (12):

- Primary infection in case of demonstration of seroconversion during pregnancy or presence of CMV low-avidity IgG and specific IgM in the first trimester of gestation;
- Non-primary infection in case of presence of IgG before pregnancy or IgG without IgM within the first trimester of gestation or 4-fold or greater rise in IgG titer in paired samples.

As a regional referring canter, we received patients from a vast area who had already been investigated in local laboratories using different cut offs. Routine repetition of maternal serology was deemed unethical.

Patients were classified as symptomatic at onset if presenting with one or more of the following: intrauterine growth restriction (IUGR), hepatomegaly, splenomegaly, petechiae, thrombocytopenia (<100,000 platelets/mm^3^), elevated serum transaminase levels, jaundice with conjugated hyperbilirubinemia, central nervous system (CNS) involvement (as denoted by microcephaly [head circumference <2 SD below the mean for age and birth weight], seizures, lethargy and/or, poor suck, neuroimaging abnormalities consistent with CMV infection detected by cranial ultrasound [US] and/or Magnetic Resonance Imaging [MRI] and Computed Tomography [CT] in past years (13) such as calcifications, neuronal migration disorders, cerebral, and cerebellar volume loss, ventriculomegaly, white matter disease, ophthalmological abnormalities detected by funduscopic examination or sensorineural hearing loss (SNHL) detected by Brainstem Auditory Evoked Responses [BAERs]). SNHL was defined as a threshold >20 dBnHL for pure tones, confirmed at two consecutive BAERs, and after exclusion of middle ear disorders. Patients were defined as asymptomatic if free from all signs listed above soon after birth. A lumbar puncture was part of the standard initial evaluation of all congenitally infected neonates until December 2010; subsequently it was performed only in symptomatic ones (viral detection was performed with the same methods described before for urine and blood).

Other causes of congenital infection, including toxoplasmosis, rubella, herpes simplex, and syphilis were ruled out.

Neonates with CNS involvement were treated with intravenous ganciclovir (GCV) or oral valganciclovir (VGCV) for at least 6 weeks, according to guidelines at the time of diagnosis (i.e., valganciclovir after 2015) (14, 15), after informed consent from parents or legal guardians was obtained. The follow-up was scheduled for 6 years in case of asymptomatic infection and longer for symptomatic one, based on clinical need. Data regarding timing and type (primary vs. non-primary) of maternal infection, neonatal and follow-up evaluations (physical, neurodevelopmental, audiological and ophthalmological assessments) were prospectively collected during periodic controls. Data were recorded on a standardized database.

The study protocol matched the standard care applied in our center to all infants with cCMV infection. Only infants followed for at least 12 months were included. Infants with no proof of cCMV infection or with other congenital infections or other chronic concomitant diseases were excluded from the study.

The Ethics Committee of our Institution (Comitato Etico “Carlo Romano,” Università Federico II di Napoli) approved this study (protocol number 274/16).

### 2.2. Ophthalmological evaluation

Fundus examination and cycloplegic refraction were performed. A single instillation of tropicamide hydrochloride was adopted to induce cycloplegia.

According to International Classification of Diseases, 10th Revision, vision was classified as normal, moderate impairment, severe impairment and blindness. Normal vision was defined as best corrected visual acuity (BCVA) equal or better than 20/70, measured with optotypes, or the ability to fix and to follow a near object for pre-verbal or no cooperating children. Moderate visual impairment was characterized by BCVA equal or better than 20/200 or by the presence of fixation but no following near object. Severe impairment was defined as BCVA worse than 20/200 or absence of fixation and following. Finally, BCVA worse than 20/400 in the better eye denotes blindness.

Motility and anterior segment examination were performed in all infants. Patients with fundus and/or visual function abnormalities underwent visual evoked potentials (VEPs). VEPs analyzed occipital cortex response to flash and/or pattern reversal stimulus.

The first ophthalmological evaluation was performed within the first 4 weeks of life in order to classify the type of infection and as indication to start antiviral therapy. In both symptomatic and asymptomatic patients, a complete ophthalmological evaluation, including fundus examination, motility and visual acuity assessments was repeated at 3 months of age, then every 6 months up to 2 years and every year thereafter. This schedule was modified depending on specific ocular findings.

### 2.3. Data analysis

All data analyses were performed using SPSS (Statistics for Windows, Version 21.0). Numerical variables were described using mean ± standard deviation (SD) or median with range [min; max] while categorical variables were summarized using absolute frequencies and percentages. Between-groups differences were, accordingly, assessed by the unpaired *T*-test or the Mann-Whitney *U*-tests and the Chi square test, or the Fisher exact test if appropriate. All tests were 2-tailed and *p* < 0.05 were considered statistically significant.

## 3. Results

A cohort of 250 congenitally infected children was analyzed and the disease onset was symptomatic in 123 (49%) infants and asymptomatic in 127 (51%) ones. Demographical data of the study population are shown in Table 1. Median duration of follow-up was longer for symptomatic infants (4.7 vs. 2.9 years, *p* < 0.001). Symptomatic and asymptomatic patients were compared according to viral load in blood, urine and cerebrospinal fluid (CSF) at onset, as shown in Table 2. The evaluation of cerebrospinal fluid (CSF) was available for 52 asymptomatic and 84 symptomatic patients.

**Table 1:** Demographical data of the population in study.

	Symptomatic *N* = 123	Asymptomatic *N* = 127	*p*-value
Sex			*0*.*129*^a^
M	59 (48%)	71 (56%)	* *
F	64 (52%)	56 (44%)	* *
Gestational age (weeks) Mean ± SD (min–max)	36 ± 4 (24–42)	38 ± 2 (31–42)	***<0***.***001***^b^
Birth weight (grams) Mean ± SD (min–max)	2463.33 ± 891.05 (440–4350)	3027 ± 547.53 (1310–4540)	***<0***.***001***^b^
Follow-up duration (years) Median (min–max)	4.7 (1–13.3)	2.9 (1–10.3)	***<0***.***001***^b^
Type of maternal infection			***<0***.***001***^a^
- Primary	48 (39%)	82 (64%)	*** ***
- Non-primary	40 (33%)	20 (16%)	*** ***
- Not available data	35 (28%)	25 (20%)	*** ***

^a^ *X*^2^.

^b^ *t*-Student.

Statistically significant *p*-values are in bold.

**Table 2:** Characteristics of viral load at onset in symptomatic and asymptomatic patients.

	Symptomatic	Asymptomatic	*p*-value
CMV DNA in blood (positive vs. negative)	75/123 (61%)	61/127 (48%)	*0*.*224*^a^
Blood viral load (copies/ml; min–max)	58,015	4,287	***0.025*** ^b^
	(234–1,134,000)	(178–49,600)	*** ***
Urine viral load (copies/ml; min–max)	10,369,737	4,814,068	*0*.*306*^b^
	(1,000–572,000,000)	(399–111,000,000)	* *
CMV DNA in CSF (positive vs. negative)	13/84 (15%)	0/52 (0%)	***<0***.***001***^a^

^a^ *X*^2^.

^b^ *t*-Student.

CSF: cerebrospinal fluid.

Statistically significant *p*-values are in bold.

Characteristics of the symptomatic infants with and without ocular involvement at birth and during follow-up are shown in Table 3. No difference was found between the two groups when compared according to viral load in blood and urine, as shown in Table 3. Furthermore, also the viral load in CSF (available for 13 patients, 5 with chorioretinitis) did not show statistically significant differences (*p* 0.453).

**Table 3:** Characteristics of the symptomatic infants with ocular involvement at birth and during follow-up.

	Ocular involvement *N* = 16	Non ocular involvement *N* = 107	*p*-value
Sex			0.285^a^
M	10 (62%)	58 (54%)	
F	6 (38%)	49 (46%)	
Gestational age (weeks) Median (min–max)	38 (34–42)	38 (24–41)	0.401^b^
Birth weight (grams) Median (min–max)	2547 (1250–3990)	2580 (440–4350)	0.623^b^
Follow-up duration (years) Median (min–max)	7.1 (1.1–11.1)	4.5 (1–13.3)	0.140^b^
Type of maternal infection			0.429^a^
Primary	4 (25%)	44 (41%)	
Non-primary	7 (44%)	33 (31%)	
Non available data	5 (31%)	30 (28%)	
Blood viral load (copies/ml; min–max)	36,938	61,257	0.701^b^
	(268–228,150)	(234–1,134,000)	
Urine viral load (copies/ml; min–max)	2,322,076	11,507,790	0.570^b^
	(4,844–20,000,000)	(590–572,000,000)	

^a^ *X*^2^.

^b^ *t*-Student.

Funduscopic abnormalities were identified in neonatal period in 16/123 (13%) symptomatic infants and in none of the infants without other clinical and instrumental abnormalities at birth and therefore classified as asymptomatic ones (*p* < 0.001).

Chorioretinitis was the most common findings (10/16 cases, 62.5%), while the other 6 patients presented with chorioretinal scars since birth. Chorioretinal involvement was bilateral in 4 patients (25%) and monolateral in the others. All but one received antiviral therapy with ganciclovir or valganciclovir, according to guidelines of the time of diagnosis. The only patient who did not receive therapy was a lost to follow up from diagnosis (at birth) to approximately one year of age. She was diagnosed as cCMV but did not complete onset evaluation (especially fundoscopy in the first month of life showed not clear evidence of a small retinal scar). When she was evaluated again around one year of life, she had a stable scar at fundoscopy and an otherwise normal MRI findings without any other signs of congenital infection. At last evaluation she was three years old and had a stable scar without visual impairment and other audiological and neurological problems.

All infants with ocular involvement at birth had MRI abnormalities, such as periventricular calcifications, dilatation of the lateral ventricles up to the ventriculomegaly (but none needed a derivation), thinning of the corpus callosum, micro polygyria, white matter abnormalities, hippocampal dysplasia. During the follow up period in the group of symptomatic children with ocular involvement 7 of them showed a normal psychomotor development. Eight of them developed severe psychomotor delay associated with seizures in 5 cases. The remaining one showed speech delay and mild psychomotor delay. Considering the hearing function in this subgroup, SNHL was diagnosed in 8 children at birth (bilateral in 6 cases) and confirmed at last evaluation in 5 patients (three of them needed a prothesis).

After the first evaluation, no patient either asymptomatic or with cCMV manifestations developed new retinal lesions. Throughout the follow up period, no asymptomatic infant was diagnosed in visual impairment while it was detected in 5/16 (31.5%) symptomatic infants (*p* < 0.001 vs. asymptomatic ones). Children presenting with multiple lesions or bigger ones in critical regions more often presented with low vision at last ophthalmological evaluation. They also presented abnormal VEPs and optic nerve atrophy more frequently. None of the children presented with nystagmus while strabismus was detected in 2 children. All patients with severe ophthalmological outcome had also significant neurological impairment.

Among symptomatic patients, chorioretinitis was associated with pathological findings in prenatal ultrasonography (*p* 0.05) and other signs of central nervous system (CNS) involvement at birth (in 50% and 44% of patients respectively, *p* 0.046). No correlation was found with the type of maternal infection during pregnancy and pathological neuroimaging.

Detailed characteristic of the symptomatic infants with ocular involvement at birth and during follow-up are described in Table 4.

**Table 4:** Characteristics of the symptomatic infants with ocular involvement at birth and during follow-up.

Patient	Ocular findings at birth	Neuroimaging findings (MRI)	Other signs at birth	Hearing function	Antiviral therapy	Maternal infection	Last ophthalmological evaluation (age)	Neurological outcome
1	R: chorioretinitis in the paramacular area, inferior temporal sector, about ½ of the papillary diameter	Periventricular calcifications	no	SNHL (R&L 60 dB) at birth; normal at last evaluation	GCV 6 weeks	MPI in 2nd trimester	R: chorioretinal scar; normal vision (11.3 years)	normal
2	L: small chorioretinal scar	Ex-vacuo dilatation of the trigons and temporal horns of the lateral ventricles; thinning of the corpus callosum; subcortical and temporo-mesial atrophy	IUGR, petechiae, thrombocytopenia, hepatomegaly	SNHL (R 70 dB; L 40 dB) at birth; normal at last evaluation	GCV 6 weeks	ND	L: small chorioretinal scar; normal vision (8.6 years)	Speech delay; mild psychomotor delay
3	R: small focus of paramacular chorioretinal scar	Temporo-polar gliotic lesion; thinning of the corpus callosum	no	normal	GCV 6 weeks	NPI in 1st trimester	R: small chorioretinal scar; normal vision (8.2 years)	normal
4	R: small chorioretinal scar between the disc and the macula	Perivascular gliosis, ventricular asymmetry and dilatation of the temporal horns of the lateral ventricles	petechiae, thrombocytopenia, hepatosplenomegaly	normal	GCV 6 weeks	NPI in 1st trimester	R: small chorioretinal scar; abnormal VEPs; normal vision (9.9 years)	psychomotor delay; seizures
5	L: chorioretinitis below the inferior temporal branch	Mild ventriculomegaly	no	normal	VGC 6 months	NPI in the 3rd trimester	L: small chorioretinal scar; normal vision (7.1 years)	normal
6	R: chorioretinitis in paramacular region	Micro polygyria; mild ventriculomegaly, thinning of the corpus callosum	no	SNHL (no response R) at birth; (R no response; L 40 dB) at last evaluation	VGC 6 months	NPI in the 1st trimester	R: chorioretinal scar; abnormal VEPs; low vision (4.9 years)	Severe psychomotor delay; seizures
7	L: chorioretinal scar in the posterior pole	Periventricular WM abnormalities	no	normal	VGC 6 months	MPI in the 2nd trimester	L: chorioretinal scar; normal vision (9.5 years)	normal
8	R: multiple lesions involving the lower sector. L: large area of chorioretinitis interesting the posterior pole temporally to the macula	Several periventricular calcifications; WM abnormalities; micro polygyria and agyria; mild ventriculomegaly; hippocampal dysplasia	Microcephaly, seizures, cholestatic hepatitis	SNHL (R 50 dB; L 60 dB) at birth; normal at last evaluation	GCV 6 weeks	NPI in the 1st trimester	R: chorioretinal scars; abnormal VEPs; low vision (2.2 years)	Severe psychomotor delay; seizures
9	R&L: multiple foci of chorioretinitis	Multiple small periventricular cysts; mild ventriculomegaly	IUGR, microcephaly, severe SNHL	SNHL (R&L no response) prothesis	VGC 6 months	ND	R&L: chorioretinal scars; abnormal VEPs; low vision (2.9 years)	Severe psychomotor delay
10	R&L: multiple foci of chorioretinitis; pale optic disc	Micro polygyria and pachygyria; several subependymal calcifications; mild ventriculomegaly; corpus callosum hypoplasia; cisterna magna	IUGR, microcephaly, petechiae, thrombocytopenia; seizures	SNHL (R no response) prothesis	GCV 6 weeks	NPI in the 2nd trimester	R&L: chorioretinal scars; abnormal VEPs; low vision (2.1 years)	Severe psychomotor delay; spastic paresis; seizures
11	R: widespread chorioretinal involvement in the inferior temporal and nasal sectors and macular lesion; L: chorioretinitis in the lower sector	Several periventricular calcifications; WM abnormalities	IUGR, prematurity, microcephaly, hepatosplenomegaly, petechiae, seizures, SNHL	SNHL (R&L no response) prothesis	GCV 6 weeks	ND	R: chorioretinal scars; microphthalmia, exotropia; abnormal VEPs; normal vision (8.6 years)	Severe psychomotor delay; spastic paresis
12	R: chorioretinal scar in peripapillary area	Multiple small periventricular cysts; WM abnormalities	no	normal	VGC 6 months	MPI in the 2nd trimester	R: chorioretinal scar; normal vision (1.7 years)	normal
13	R: small area of chorioretinitis in paramacular region	Diffused WM abnormalities; mild ventriculomegaly	IUGR, prematurity, microcephaly, seizures	SNHL (L no response at birth and at last evaluation)	VGC 6 months	NPI in the 1st trimester	R: chorioretinal scar; exotropia; abnormal VEPs (1.1 years)	Spastic hemiparesis R; psychomotor delay
14	L: area of chorioretinitis in paramacular region	Tetra ventricular hydrocephalus; several calcifications; hypoplasia of the cerebellar worm; mega cisterna magna; diffused WM abnormalities	IUGR, prematurity, petechiae, tetra ventricular hydrocephalus	Normal at birth; SNHL (R&L 40 dB) at last evaluation	GCV 6 weeks	MPI in the 1st trimester	L: chorioretinal scar; abnormal VEPs; low vision (4.8 years)	Severe psychomotor delay; spastic paresis; seizures
15	R: area of chorioretinitis in paramacular region	WM abnormalities	Prematurity	normal	GCV 6 weeks	ND	R: small chorioretinal scar; normal vision (7.1 years)	normal
16	R: not clear evidence of small chorioretinal scar	Asymmetric signal by the optic nerves (related to chorioretinal damage)	IUGR	normal	no	ND	R: small chorioretinal scar; normal VEPs (2.9 years)	normal

MRI, magnetic resonance imaging; GCV, ganciclovir; IUGR intrauterine growth restriction; L, left; R, right; SNHL, sensorineural hearing loss; VGCV, valganciclovir; WM, white matter; MPI, primary infection; NPI, non-primary infection; ND, not definable; VEPs, visual evoked potential.

## 4. Discussion

Our data show fundoscopic abnormalities in 13% of symptomatic cCMV infants (16/123), somewhat less than what reported by the most recent studies (14/77 in Jin H et al. (8) that is 18% and 7/18 in Capretti MG et al. (9) that is 39%. Our data is more similar to the one reported by Boppana SB et al. (16) in another series of patients (10% of symptomatic infants, 4/39).

Among ocular findings, chorioretinitis was the most common (62.5%). As previously described (8) the posterior segment of the eye was more frequently affected by congenital CMV infection, resulting is chorioretinal scars at birth. It was suspected that the virus’s high tropism to rapidly developing neurons during the first trimester of pregnancy is responsible for the neuron-related damages as seen in retina scar (8). Unfortunately, data about time of maternal infection was available for a very small number of patients in our cohort so it was not evaluated.

The initial diagnosis of chorioretinitis was associated with pathological findings in prenatal ultrasonography and the presence of other signs of central nervous system (CNS) involvement at birth. These data could suggest that an early ophthalmological evaluation is highly recommended in neonates suspected with symptomatic cCMV infection. In fact, an ophthalmologic involvement seems to be more frequent in infants with more severe disease.

Long-term visual impairment reported in 31.5% of patients with a chorioretinal scar at birth and was related with multiple lesions or bigger ones in critical regions, frequently associated with abnormal VEPs and optic nerve atrophy. Moreover, SNHL was also detected in symptomatic patients with ophthalmological impairment as previously described (8) and the combination of these two sensory disorders would potentially make communication with these patients a challenge.

Giannattasio et al, 2017 (17) showed that clinical findings at birth and a severe cCMV disease were not affected by the type of maternal infection. In this present study we confirmed no correlation between ocular involvement and type of maternal infection as reported by Capretti MG et al. (9).

One patient in our cohort was temporarily lost at follow up and did not receive antiviral therapy despite a chorioretinal scar. When she was then seen at the age of three, she had a stable scar without visual impairment and other audiological and neurological problems. Though reported in a single case, these findings question the efficacy of current drug regimen in isolated eye involvement and should be evaluated in a larger population.

Unlike previous reports of late-onset retinal lesions and of reactivations of chorioretinitis during childhood was previously described (8, 18, 19), we did not identify new lesions during the follow-up period neither in the asymptomatic nor in the symptomatic group. In this respect, we expand on a larger cohort similar observation by Capretti MG et al. (9). The long follow-up period, particularly in the symptomatic group, is also a point of strength of the present paper. We suspect that after birth the eye is less susceptible to viral damage, as it happens for brain structures that can be affected during the fetal life and not after birth.

In our cohort of 250 cCMV infected infants no ophthalmological abnormalities were detected in asymptomatic ones, nor at onset nor during follow-up. In previous reports, the presence of ophthalmological abnormalities in infected but asymptomatic babies is controversial. A possible explanation may reside in the role of a normal brain MRI in the definition of asymptomatic patient. This criterion is adopted in our study and by Capretti but not in the papers by Coats DK and Jin H et al. (5, 8) and may also justify a better visual prognosis in these patients. It is noteworthy that when this strict definition is followed the audiological prognosis is also favorable (20).

Our study has its limitations. Although we provide data from a large series of infected cCMV babies, only 16 of them had eye involvement and a larger numerosity may reinforce our findings. Also, the great number of mothers who had incomplete CMV screening during pregnancy impeded us to prove a correlation between ocular involvement and the type and time of maternal infection. Finally, although ocular involvement from other perinatal infections was excluded in our series, we did not rule out more uncommon causes of neonatal chorioretinitis.

## 5. Conclusions

Chorioretinitis is a possible finding in neonates with symptomatic (but not in truly asymptomatic) cCMV infection. These babies deserve an early ophthalmological evaluation. A careful follow up may be necessary to those with lesions at the initial examination.

## Data Availability

The raw data supporting the conclusions of this article will be made available by the authors, without undue reservation.
